# Supplementary material for: Metapopulation epidemic models with heterogeneous mixing and travel behaviour
Source: Theor Biol Med Model. 2014 Jan 13;11:3. doi: 10.1186/1742-4682-11-3 (PMC3909360; doi:10.1186/1742-4682-11-3)
Supplement: Additional file 1 — Approximations and series expansion used for estimating the invasion threshold parameter for a proportionate and an assortative social system. [file 1742-4682-11-3-S1.pdf]

Additional File 1

Metapopulation epidemic models with heterogeneous mixing and travel behaviour

A. Apolloni, C. Poletto, J.J. Ramasco, P. Jensen, V. Colizza

Series expansion for the proportionate mixing case

In this section we provide the details of the series expansion in the quantities z_1, z_2 and π_1, π_2 that allow for recovering the approximate results for R_* of Eqs. (23) and (24).

For the limit $\eta \to 0$, the epidemic size of Eq. (22) can be approximated by

$$z_1 \simeq 2\,\frac{(R_0-1)}{R_0^2}\left(1+\eta^2\,\frac{(1-\alpha)}{\alpha\,R_0}\right),$$
$$z_2 \simeq 2\,\eta\,\frac{(R_0-1)}{R_0^2}. \tag{A.1}$$

While the extinction probabilities, which are the solutions for Eq. (11), are

$$\pi_1 \simeq \frac{1}{R_0}\left(1-\eta^2\,\frac{(1-\alpha)\,(R_0-1)}{\alpha}\right),$$
$$\pi_2 \simeq 1-(R_0-1)\,\eta. \tag{A.2}$$

We combine these expressions into Eq. (15) and we keep only the first not-constant terms, recovering in this way Eq. (23).

For the case $\eta \to 1$ we recover first the solutions for Eq. (22) in the first order in $(1-\eta)$

$$z_1 \simeq \frac{2\,(R_0-1)}{R_0^2}\left(1+(1-\eta)\,\frac{(1-\alpha)}{R_0}\right)$$
$$z_2 \simeq \frac{2\,(R_0-1)}{R_0^2}\left(1+(1-\eta)\,\frac{(1-\alpha-R_0)}{R_0}\right). \tag{A.3}$$

and then the extinction probabilities become

$$\pi_1 \simeq \frac{1}{R_0}\left(1-(1-\eta)\,\frac{(1-\alpha)\,(R_0-1)}{R_0}\right)$$
$$\pi_2 \simeq \frac{1}{R_0}\left(1+(1-\eta)\,\frac{\alpha\,(R_0-1)}{R_0}\right). \tag{A.4}$$

with these expressions for $\pi_{1,2}$ and together with $z_{1,2}$ of Eq. (A.3), we find in this limit Eq. (24). As expected the first term in Eq. (24) is the homogenous solution R_*^h, while the second is liner in $1-\eta$.

Series expansion for the assortative mixing case

For the case of assortative mixing scenario the contact matrix and next generation matrix are more complex and need to be expanded before handling Eq. (5) and Eq. (11). We first consider the series expansion in ϵ of the basic reproductive number, which up to second order term reads

$$R_0 \simeq \frac{\beta\, q_1}{\mu} \left(1 + \frac{\epsilon}{\alpha} + \frac{\epsilon^2}{(1-\alpha)\,\alpha}\right). \tag{A.5}$$

We go up to the second order because it is necessary to find the leading terms for R_* in the limit $\eta \to 0$ and $r \to 0$. However, most of calculations are performed up to first order in ϵ only. The contact matrix can be written in this limit as

$$\boldsymbol{C} \simeq q_1 \times \tag{A.6}$$
$$\begin{pmatrix} \frac{1}{\alpha} - \frac{\epsilon^2}{(1-\alpha)\,\alpha^2\,(1-\eta)} & \frac{\epsilon}{\alpha\,(1-\alpha)}\left(1+\frac{\epsilon}{\alpha}\right) \\ \frac{\epsilon}{\alpha\,(1-\alpha)}\left(1+\frac{\epsilon}{\alpha}\right) & \frac{\eta}{1-\alpha} - \frac{\epsilon\,(\alpha-\eta\,(1-\alpha))}{\alpha\,(1-\alpha)^2} + \frac{\epsilon^2\,(\eta\,(1-\alpha)-\alpha)}{\alpha^2\,(1-\alpha)^2\,(1-\eta)} \end{pmatrix}.$$

While the next generation matrix becomes

$$\boldsymbol{R} \simeq R_0 \times \tag{A.7}$$
$$\begin{pmatrix} 1-\frac{\epsilon^2}{(1-\alpha)\,\alpha\,(1-\eta)} & \frac{\epsilon\,(\alpha+\epsilon)}{(1-\alpha)\,\alpha} \\ \frac{\epsilon\,(\alpha+\epsilon)}{\alpha^2} & \eta+\epsilon\left(\frac{\eta}{\alpha} - \frac{1}{1-\alpha}\right)+\epsilon^2\,\frac{\eta\,(1-\eta)-\alpha\,(1+\eta-\eta^2)}{(1-\alpha)\,\alpha\,(1-\eta)} \end{pmatrix}.$$

The solution of the epidemic size for the two groups is obtained by considering first the behaviour of (5) around the epidemic threshold, $R_0 \approx 1$, and then expanding the solutions up to first or second order with respect to ϵ. In this way, the solutions can be written as a sum of terms in order zero, one and two in ϵ, $z_{1,2} = z_{1,2}^0 + \epsilon\, z_{1,2}^1 + \epsilon^2\, z_{1,2}^2$. As before and for simplicity, we consider two limits regarding the contact rate of individuals of the two types: $\eta \to 1$ and $\eta \to 0$. Note, however, that the limit has to satisfy the condition $\epsilon < \eta\,(1-\alpha)$.

For the case $\eta \to 0$, by taking into account the matrices of Equations (A.7) and (A.6), we can solve Eq. (5) obtaining for the epidemic sizes

$$\begin{aligned} z_1 &\simeq \frac{2\,(R_0-1)}{R_0^2} \\ &\quad - \epsilon^2\,\frac{2\,(R_0-1)\,(R_0-2)\,(1+\eta\,(R_0+1))}{\alpha\,R_0^2\,(1-\alpha)}, \\ z_2 &\simeq \epsilon\,\frac{2\,(R_0-1)\,(1+\eta\,R_0)}{R_0\,(1-\alpha)}, \end{aligned} \tag{A.8}$$

and for the extinction probabilities $\pi_{1,2}$

$$\begin{aligned}\pi_1 &\approx \frac{1}{R_0} - \epsilon^2\,\frac{(R_0-1)\,(1+\eta\,(R_0+1))}{\alpha\,R_0\,(1-\alpha)},\\ \pi_2 &\approx 1-\epsilon\,\frac{(R_0-1)}{1-\alpha}\,(1+\eta\,R_0).\end{aligned} \tag{A.9}$$

The terms of the type 2 individuals appear in Eq. (15) within the product $(1-\pi_2)\,z_2$. In zero order in ϵ, $\pi_2^0 = 1$ and $z_2^0 = 0$. Therefore, the only terms contributing to R_* in order ϵ^2 of the type 2 individuals are the first order terms π_2^1 and z_2^1. For this reason and for clarity, we have shown in Eqs. (A.8) and (A.9) the first order terms for π_2 and z_2 alone. The same cannot be said for the terms of type 1 individuals and so the expansions in π_1 and z_1 are taken up to second order. Inserting the results of Eqs. (A.8) and (A.9) into Eq. (15), we find the solution of Eq. (25).

In the limit $\eta \to 1$, the solutions for the epidemic sizes in single populations reads

$$\begin{aligned}z_1 &\simeq \frac{2\,(R_0-1)}{R_0^2} - \epsilon\,\frac{2\,(R_0-2)}{\alpha\,R_0^2},\\ z_2 &\simeq \frac{2\,(R_0-1)}{R_0^2} + \frac{2\,(R_0-2)}{R_0^2}\left((1-\eta)-\frac{\epsilon}{\alpha}\right).\end{aligned} \tag{A.10}$$

The analytical result above points out a peculiar feature of the assortative system this limit condition: small but non-zero outbreaks are possible even for $R_0 = 1$. Such outbreaks have size proportional to a combination of ϵ and $1-\eta$ which are vanishing quantities [1]. The extinction probabilities in this case are

$$\begin{aligned}\pi_1 &\simeq \frac{1}{R_0}\left(1-\frac{\epsilon}{\alpha}\right),\\ \pi_2 &\simeq \frac{1}{R_0}\left(1+(1-\eta)-\frac{\epsilon}{\alpha}\right).\end{aligned} \tag{A.11}$$

With these expressions for $z_{1,2}$ and $\pi_{1,2}$, we find Eq. (26). In this case we include the linear terms leaving out the sub-leading terms of order ϵ^2 and $\epsilon\,(1-\eta)$.

References

[1] P van den Driessche and James Watmough. Reproduction numbers and sub-threshold endemic equilibria for compartmental models of disease transmission. *Math Biosci*, 180:29–48, November 2002.
